# Renal and electrolyte complications in eating disorders: a comprehensive review

**DOI:** 10.1186/s40337-023-00751-w

**Published:** 2023-02-20

**Authors:** Leah Puckett

**Affiliations:** 1 ACUTE Center for Eating Disorders and Severe Malnutrition Denver, Denver, CO USA; 2 Denver Health Medical Center, 723 Delaware Street, Fl. 3, Denver, CO 80204 USA; 3 University of Colorado School of Medicine Aurora, Aurora, CO USA

**Keywords:** Eating disorders, Anorexia nervosa, Binging, Purging, Kidney failure, Renal disease, Electrolytes

## Abstract

Eating disorders are psychiatric disorders with significant and widespread medical complications, including renal disorders. Renal disease is not uncommon in patients with eating disorders but is often unrecognized. It includes both acute renal injury and progression to chronic kidney disease requiring dialysis. Electrolyte abnormalities including hyponatremia, hypokalemia, and metabolic alkalosis are common in eating disorders and vary depending on whether patients engage in purging behaviors. Chronic hypokalemia due to purging in patients with anorexia nervosa-binge purge subtype or bulimia nervosa can lead to hypokalemic nephropathy and chronic kidney disease. Additional electrolyte derangements are seen during refeeding, including hypophosphatemia, hypokalemia, and hypomagnesemia. Patients can also develop Pseudo-Bartter’s syndrome which leads to edema and rapid weight gain in patients who cease purging behavior. Clinicians and patients should be aware of these complications in order to provide education and early detection and prevention.

## Plain English summary

Eating disorders are common and serious mental health disorders with frequent medical complications. This review discusses some of the problems with kidney function and electrolyte abnormalities that occur in patients with eating disorders. Common eating disorders discussed are anorexia nervosa and bulimia nervosa. Anorexia nervosa involves eating very small amounts of food and may include intentional vomiting or misuse of medications called laxatives or diuretics. These behaviors can cause decreased blood flow to the kidneys and subsequent kidney failure, as well as problems with important electrolytes in the body including potassium and sodium. These can lead to very serious problems, including death. Patients dealing with these issues should be seen by their doctor and potentially hospitalized for treatment.

## Background

Eating disorders (ED) contribute substantially to widespread organ dysfunction [1], including disorders of kidney function. The kidneys perform vital functions throughout the body, including acid–base and mineral homeostasis, metabolism and excretion of substances, and maintenance of blood pressure and volume status [2]. Risk factors for renal disease in ED are related to the type of ED and specific behaviors engaged in by the patient. Anorexia nervosa-restricting subtype (AN-R), where weight loss is achieved through dieting and fasting, has different implications for renal disease than patients with anorexia nervosa-binge purge subtype (AN-BP), in which patients restrict caloric intake and engage in recurrent binge eating episodes and compensatory purging behaviors (laxative, diuretic abuse, and/or self-induced vomiting) [3]. In bulimia nervosa (BN), behavior and therefore renal implications are similar to AN-BP, however, the patients do not restrict caloric intake. Therefore, similar renal complications are seen in AN-BP and BN.

Abnormalities in kidney function and electrolyte disturbances are frequently seen in patients with ED, including acute kidney injury (AKI) and chronic kidney disease (CKD) [4], most often related to chronic hypokalemia (serum potassium < 3.5 mEq/L) and volume depletion [5]. AKI is a “heterogeneous group of conditions characterized by a sudden decrease in glomerular filtration rate (GFR) followed by an increase in serum creatinine concentration or oliguria [low urine output]” which can lead to serious complications including volume overload and electrolyte abnormalities and can progress to CKD [6]. Mechanisms of kidney disease in ED are complex and infrequently studied and thus incompletely understood [7, 8]. Electrolyte abnormalities including hypokalemia, hyponatremia (serum sodium < 135 mEq/L), and acid–base disturbances are common and their occurrence and severity generally correspond to engagement in purging behaviors [9, 10].

This review provides a comprehensive description of the current understanding of the pathophysiology, diagnosis and treatment of renal and electrolyte disturbances observed in patients with an ED. A robust qualitative review of the literature over the last 10 years was performed via electronic database search given the lack of large, controlled trials available for this subject matter. Earlier articles were included when relevant.

## Measuring kidney function in eating disorders

Accurately evaluating kidney function in patients with an ED poses certain challenges and limitations. To better understand these limitations, it is helpful to discuss general principles of assessing kidney function. Kidney function is evaluated using glomerular filtration rate (GFR), which provides an overall assessment of kidney function and is helpful in diagnosing, staging and managing kidney disease [2]. Decreased GFR correlates with degree of pathology in CKD and is observed prior to manifesting symptoms of kidney disease; low GFR is by definition kidney failure [2]. GFR measures the filtration rate of the glomerulus, a collection of small blood vessels (capillaries) adjacent to the kidney nephrons, which filter plasma [2]. Measured GFR (mGFR) is indirectly obtained using the rate of clearance of an exogenous substance (often a radioisotope) which is complex and difficult to perform. Thus, in clinical practice we generally use estimated GFR (eGFR), based on clearance of an endogenous rather than exogenous substance–most commonly creatinine, as it is widely available and affordable. Creatinine is generated by muscle mass or dietary intake, usually from animal protein products [2]. Therefore, eGFR methods that utilize creatinine less accurately reflect kidney function in patients with very high or low muscle mass, low protein intake, and hypovolemia compared to healthy individuals [2, 11, 12] as creatinine can be falsely low in AN patients [12].

In such populations, another potential marker of eGFR is cystatin C, a protein that is less affected by muscle mass and dietary intake than creatinine [5, 12] and is often used to measure kidney function in the pediatric population [12]. Somewhat surprisingly, however, a study in adolescents with AN found cystatin C did not accurately reflect eGFR, as it was normal in all study patients despite 35% of subjects having a mGFR under 90 mL/min/1.73 m^2^. Authors postulate perhaps cystatin C is a less reliable marker earlier in the progression of kidney disease [12]. In that study the optimal eGFR was calculated using Cockroft-Gault formula [12]. However, Cockroft-Gault has been found in other studies in patients with AN or malnutrition to overestimate GFR; thus it seems both creatinine and creatinine-based equations are not necessarily reliable in malnourished patients [5]. Further complicating accurate GFR assessment, isotopic methods of measuring GFR may not be accurate at low BMI [13].

Further studies are needed to optimize evaluation of kidney function in this population. Use of currently available methods can be employed and are helpful for establishing baseline kidney function for comparison over time. However, these methods should be interpreted with caution and awareness of their limitations.

## Prevalence of kidney disease in eating disorders

There is a lack of large population-based studies regarding AKI and progression to CKD in patients with AN, and many of the available studies do not differentiate between subtypes of AN, so it is difficult to define the prevalence of kidney disease in this population. However, some data estimates > 5% of patients with AN will develop end-stage renal disease (ESRD) after 21 years (AN subtype not specified) [7, 11]. ESRD refers to irreversible loss of kidney function expected to result in death within days or weeks from complications such as hyperkalemia (serum potassium levels > 5 mEq/L) or pulmonary edema without intervention with dialysis or renal transplant [14]. Dialysis has significant effects on quality of life, and patients on dialysis have lower life expectancy, primarily due to increased risk of cardiovascular disease and infection [14]. The rate of dialysis in patients with AN is unknown, though there are case series and case reports of patients with AN who developed ESRD and underwent dialysis, including one patient with longstanding AN-BP who began peritoneal dialysis with subsequent death from sepsis [15, 16].

One retrospective study of hospitalized adolescents with a relatively new diagnosis of AN (subtype not specified) found 37% of patients had impaired kidney function. Similarly, a secondary data analysis of 120 adolescents and young adults hospitalized with medical instability from AN (subtype not specified) or atypical anorexia nervosa (AAN) found 33% of patients had impaired kidney function; risk factors in this study associated with renal impairment included rapid weight loss and severe bradycardia [17]. While these studies do not specify subtype of AN, it is very likely that most of the renal disease observed in these patients was related to effects of purging behaviors, and it is important to note that both laxative abuse and self-induced vomiting pose a significant high risk for renal failure.

However, there is some literature describing renal impairment in AN-R. One retrospective cross-sectional study of hospitalized adolescent patients with severe malnutrition from AN-R found 72% had impaired kidney function, including 59% with eGFR 89–60 mL/min (stage 2 mild CKD), 12% with eGFR 59–45 mL/min (stage 3A moderate CKD), and 2% with eGFR 44–30 mL/min (stage 3B moderate CKD) [8].

These studies highlight the need for early screening for kidney impairment even in younger patients with AN. Patients in whom kidney disease is found should be referred to a nephrologist (kidney specialist) for further management which may include renal biopsy to rule out kidney disease from other causes. Furthermore, such patients should be educated regarding the risks of purging behaviors [16]. One case series of 14 patients with a greater than 15 year history of AN-BP already had relatively severe and irreversible kidney disease by the time of their initial evaluation by a nephrologist [16]. Early diagnosis and intervention are important given low eGFR is a risk factor for CKD and eventual ESRD [17].

## Pathophysiology of kidney disease in eating disorders

Kidney disease in ED is complex and incompletely understood [8, 11]. While kidney disease is observed in both AN and BN, it can be challenging to clearly delineate causes attributed to type or subtype as studies often do not distinguish between these. However, mechanisms of kidney disease vary significantly based on subtype of ED and behaviors involved. Risk factors for kidney disease include nutritional status, duration of disease, chronic dehydration caused by decreased oral intake and/or purging behaviors, nephrocalcinosis (calcium deposits in the kidneys), body mass index (BMI), bradycardia, and hypokalemia [8, 15, 18].

### Kidney disease in AN-R

The driving factors for renal disease in patients with AN-R are less clear than in patients with purging behaviors. However, malnutrition itself is a risk factor for kidney disease, given adequate nutrition is necessary for optimal kidney function. Low protein intake, which can be seen in individuals with restrictive diets, leads to impaired GFR and renal plasma flow. Protein-calorie malnutrition interferes with the kidney’s ability to concentrate urine and excrete sodium and acid [11]. While some data indicate low BMI as a risk factor for kidney disease, impaired kidney function is also seen in AAN, demonstrating the potential effects of starvation on renal function even at higher weight [8, 17].

Kidney failure due to rhabdomyolysis (muscle destruction usually occurring due to over-exertion, trauma or stroke with by-products being released into the bloodstream) has been described in AN, which can be caused by hypokalemia and hypophosphatemia (serum phosphorus < 2.5 mg/dL in adults) leading to renal failure [5]. A case report described a 16-year old girl with AN (subtype not specified) who developed acute oliguric renal failure and uremia due to hypophosphatemia-induced rhabdomyolysis; she underwent dialysis and her kidney function eventually recovered [19].

While hypokalemia has been reported in approximately 14% of patients with AN-R admitted to inpatient or residential care, this finding should prompt screening for covert purging behaviors as this is a possible explanation of hypokalemia in patients with a diagnosis of AN-R [9, 11, 20]. In fact, one study found the most common etiology in patients without known eating disorders referred to a specialist to determine the cause of chronic hypokalemia was covert purging from bulimia; another plausible explanation, however, is very early complications of refeeding leading to hypokalemia.

In patients with AN-R and kidney disease not attributed to the effects of covert purging and hypokalemia, hypovolemia (decreased intravascular volume) from chronic dehydration as a result of decreased oral intake combined with bradycardia may lead to decreased renal perfusion and impairment [8, 15, 18]. This is plausible given bradycardia is a very common complication of malnutrition [9]. In addition, glomerulosclerosis (hardening of the kidney in the glomeruli) observed in AN-BP is thought to be due to ischemic (decreased blood supply from arterial blockage) changes [11], a finding that could be extrapolated to AN-R given the decreased renal perfusion described above.

### Kidney disease in AN-BP and BN

Mechanisms of kidney disease in patients with AN-BP or BN, i.e. patients who utilize purging behaviors, are better elucidated. In these patients, purging behaviors which can include self-induced vomiting, abuse of laxatives, and/or diuretics lead to electrolyte and acid–base disturbances including hypokalemia [10]. Of note, hypokalemia can also occur due to refeeding syndrome. A recent study demonstrated an association of lower nadir potassium during refeeding with binge-purge behavior, lower BMI, and low albumin [21].

Chronic hypokalemia leads to nonfatty degeneration of convoluted tubules presenting as mild changes of cytoplasmic vacuolization or extensive necrosis and sloughing of tubules [22]. “Kaliopenic nephropathy” was described in 1956 by Conn and Johnson, referring to vacuolar tubulopathy in association with chronic hypokalemia [23]. The term “hypokalemic nephropathy” was coined by Cremer and Bock in the 1970’s based on a series of renal biopsies [22, 24] of patients who eventually developed chronic renal disease [24]. Rates of hypokalemic nephropathy in patients with AN (subtype not specified) are estimated 15–20% [5]. The pathophysiology of hypokalemic nephropathy is thought to be due to “renal vasoconstriction, reduced medullary blood flow and impaired renal angiogenesis” [22]. Renal biopsies performed in patients with hypokalemia and hyponatremia over a 10-year period secondary to purging behaviors demonstrated various pathological findings, including enlarged juxtaglomerular cells, vacuoles in proximal and distal tubules, and focal lymphocytic cellular infiltration [24].

While chronic hypokalemia leads to vacuolar lesions and tubulointerstitial nephritis (TIN), a recent case report describing renal biopsy of an eating disorder patient with intentional vomiting and hypokalemia showed non-uniform progression of TIN, suggesting additional factors contributing to renal disease, such as decreased renal perfusion [25]. Biopsy in this case revealed wide deposition of crystals and calcium deposits, suggesting nephrocalcinosis which can be seen in diuretic use [25].

It is also likely that acute hypokalemia is a contributor to AKI, possibly through hypovolemia and rhabdomyolysis [23]. “Hypokalemia-induced renal failure” has been described as causative in AKI in a case report in a patient with AN-BP, the authors of which describe how hypokalemia and hypovolemia together lead to ischemic changes, inflammatory cytokines, increased vasoconstriction and decreased vasodilation [23]. These findings are concerning and demonstrate the risk of purging behaviors in both AKI and CKD, which is likely compounded when patients engage in multiple modes of purging. CKD can progress to ESRD requiring hemodialysis and is a concern even in young patients with ED, particularly patients with AN-BP or BN.

The physiologic effects of laxative abuse can alert astute providers when patients are not forthcoming regarding their behaviors. One case report described the discovery of covert laxative abuse in a patient with hypokalemic non-anion gap metabolic acidosis and impaired acidification of urine. The patient was initially diagnosed with distal renal tubular acidosis, but kidney biopsy revealed lack of changes in acid-secreting protein cells, eventually establishing laxative abuse as the cause [26].

## Review of electrolyte disturbances found in eating disorders

### Overview

Electrolyte abnormalities are frequently found in patients with ED. While electrolyte abnormalities are found in AN-R, AN-BP, and BN, they are more common in patients who purge. A retrospective study of over 1000 inpatient and residential level patients with ED reported in patients with AN-R 16.8% had metabolic alkalosis (elevated serum bicarbonate), 16% had hyponatremia, and 14.2% had hypokalemia [9]. Additionally, in one study comparing patients hospitalized with avoidant-restrictive food intake disorder (ARFID) and AN (subtype not specified), there were more than double the electrolyte abnormalities in the ARFID group compared to AN, though this finding did not reach statistical significance; nevertheless electrolyte abnormalities may be problematic in this population as well [27]. Another study cited > 50% of patients with ARFID admitted to an inpatient treatment center were vomiting regularly [28], which could explain the increased electrolyte abnormalities which would likely be similar to the AN-BP or BN population.

The retrospective study of over 1000 patients also reported data for each AN subtype and BN [9]. In patients with AN-BP, 33.3% had metabolic alkalosis, 17% had hyponatremia and 42.4% had hypokalemia. In patients with BN, 23.4% had metabolic alkalosis, 8.5% had hyponatremia, and 26.2% had hypokalemia. Another retrospective study reported frequency of medical complications in 281 ED patients with AN (subtype not specified) and ideal body weight (IBW) < 65% admitted to an inpatient hospital level of care for patients with extreme ED. In this study, hypokalemia was the most common electrolyte abnormality on admission (33% of patients) and hyponatremia the second most common [29]. Sodium and potassium levels on admission were lower for patients with AN-BP than AN-R, not surprisingly [29].

Refeeding syndrome, a complex series of physiological changes occurring when glucose is re-introduced during refeeding after prolonged starvation, also leads to abnormalities of electrolytes and fluid balance in patients with ED and in severe cases may lead to death [30]. Common electrolyte abnormalities include hypophosphatemia, hypomagnesemia (serum magnesium < 1.46 mg/dL), hypokalemia, acute thiamine deficiency which can result in potentially irreversible cognitive impairment, known as Wernicke’s encephalopathy, and cardiac failure from volume overload [31].

### Hypokalemia

Hypokalemia is one of the most common electrolyte abnormalities in ED. It is present in every mode of purging as potassium can be lost in emesis, stool or urine depending on whether patients are vomiting or engaging in laxative or diuretic abuse [10]. However, most of the potassium losses are actually urinary for all modes of purging [10]. Chronic purging accentuates hypokalemia as it leads to volume loss resulting in excess secretion of aldosterone, which stimulates reabsorption of sodium and excretion of potassium, a state characterized by normotensive hypokalemic metabolic alkalosis known as Pseudo-Bartter’s syndrome [10]. Metabolic alkalosis leads to bicarbonate diuresis and loss of accompanying potassium ions in the urine. Diuretic abuse also leads to loss of potassium in the urine [10].

Effects of hypokalemia on the body are extensive and potentially life-threatening. Cardiac effects are numerous and include electrocardiogram (ECG) changes such as QTc prolongation, which increases risk of life-threatening cardiac arrhythmias, sudden cardiac death, and myocardial fibrosis [10, 32]. Hypokalemia triggers torsade de pointes (a type of ventricular arrhythmia) and may therefore be an early warning sign of sudden cardiac death [33]. Neuromuscular effects of hypokalemia include ileus, constipation, and weakness [10]. Renal effects of hypokalemia include polyuria, edema, and hypokalemic nephropathy described previously [10].

Correction of potassium depends on level of severity and presence or absence of associated metabolic alkalosis. Hyperkalemia of 2.5 mEq/L or higher can be corrected more slowly with oral potassium, given patients are asymptomatic and have no ECG changes, while critically low levels of potassium less than 2.5 mEq/L require both intravenous and oral potassium [32]. Further, it is necessary to correct associated metabolic alkalosis with isotonic saline which can be given at a rate of 50–75 mL/hour in order to disrupt the activation of the renin–angiotensin–aldosterone system which perpetuates ongoing loss of potassium in the urine as described above [32].

### Hyponatremia

Hyponatremia is another common electrolyte derangement seen in ED patients. Hyponatremia can lead to nausea/vomiting, neurologic symptoms (including headaches, altered levels of consciousness, seizures, and coma), and failure of cardiac and respiratory systems [34]. The etiology of hyponatremia differs according to ED type, although it is usually multifactorial [35]. Hypovolemic hyponatremia is the most common cause of hyponatremia in patients with AN due to decreased dietary intake of salt and water or purging behaviors. Other causes include psychiatric medications, syndrome of inappropriate anti-diuretic hormone (SIADH) or excessive water intake (“water-loading”) [35]. Psychiatric medications can lead to SIADH and may also have anti-cholinergic side effects which may cause increased thirst and water intake leading to hyponatremia.

Hyponatremia in AN-R may be related to excessive water intake, impaired renal absorption of sodium, or as a side effect of psychogenic drugs [36]. Psychogenic polydipsia is compulsive water drinking associated with psychiatric illness and can be seen in AN. One case report describes a 13 year old girl who presented to the hospital with severe hyponatremia (119 mmol/L) who had been engaging in polydipsia in order to falsely elevate her body weight and developed seizures, cardiorespiratory failure, and altered mentation [34]. Another case report described a patient with AN-R who engaged in compulsive exercise and developed hyponatremia and rhabdomyolysis not thought to be due to polydipsia or purging; proposed mechanisms were likely similar to that describe in athletes including inadequate salt replacement or SIADH secretion mediated by increased creatinine kinase [37].

In cases of purging as in AN-BP and BN, hyponatremia is associated with hypovolemia and loss of sodium due to vomiting, diuretic, or laxative abuse [10]. Decreased effective arterial volume stimulates anti-diuretic hormone secretion and increased renal water absorption thus leading to hyponatremia [10]. A case report describes psychogenic polydipsia in a patient with AN-BP drinking 8–12 L of water a day and had sodium of 111 mEq/L with associated fatigue and malaise; the patient was diagnosed with dilutional hyponatremia from polydipsia based on low serum and urine osmolality [35].

Symptomatic hyponatremia from water intoxication in patients with AN with severe malnutrition can be seen with lower amounts of water ingested than would be necessary in patients with higher body weight. This is due to physiological changes occurring in AN [35]. Typically, the kidneys can excrete up to 28 L of free water a day, but in patients with AN, this process is impaired due to multiple complex factors. Among these are low protein intake which lowers urea, decreasing glomerular filtration pressure and reabsorption of sodium. Furthermore, chronically low serum osmolality can lead to reset osmostat and lower threshold for ADH release [35]. Indeed, there are reports of patients with AN drinking as little as 4–5 L of water daily developing severe hyponatremia [34, 38]. Therefore, clinicians and patients should be aware of the potential for more rapid changes in serum sodium levels in malnourished patients and associated consequences.

Consequences of hyponatremia can be devastating. Worth mentioning due to its severity is central pontine myelinolysis (CPM), a phenomenon characterized by demyelination of axons in the central pons which can lead to symptoms varying from mild confusion to paresis, coma, and death [39]. There are few studies discussing CPM in ED patients, but a recent case report describes a 24-year old patient with recurrent hyponatremia and hypokalemia due to BN who developed CPM which clinical findings of paresis, dysphagia, and dysarthria [39]. While CPM has mostly been attributed to rapid correction of hyponatremia, another recent case report described a 31-year old man with AN-BP who developed transient MRI findings of CPM in the context of rapidly changing potassium levels, hypothesized to be due to increased osmotic pressure from administered fluids for electrolyte correction [40].

Another potential long-term effect of hyponatremia relevant to patients with ED is bone disease [19]. A recent retrospective study of hospitalized adolescents with AN evaluated the association between hyponatremia and bone mineral density (BMD) and found lower DXA Z scores in patients who had a prior episode of hyponatremia. Authors hypothesize a novel mechanism of hyponatremia mediating malnutrition-related bone disease in patients with AN, a proposal supported by animal models and data in older patients [19].

Correction of hyponatremia also depends on the degree of severity. Mild hyponatremia (130–135 mEq/L) generally self-corrects once purging behaviors (if present) cease and patients resume nutritional intake. However, for sodium < 125 mEq/L, patients should be treated with a slow intravenous infusion of isotonic saline, increasing sodium no more than 4 to 6 mEq/L over a 24-h period to avoid the aforementioned CPM [32]. Severe hyponatremia < 118 mEq/L requires transfer to the intensive care unit for closer monitoring and potential renal consultation for administration of a medication called desmopressin which can help the sodium from being corrected too quickly [32].

### Metabolic alkalosis

Metabolic alkalosis is a state of volume contraction caused by dehydration often caused by purging, usually found in conjunction with hypokalemia and hypovolemic hyponatremia [9]. Dehydration leads to increased aldosterone, renin, and angiotensin and therefore increased bicarbonate resorption in the kidneys in order to maintain volume status [9]. More severe metabolic alkalosis is more often found in patients with AN-BP than AN-R. This finding along with hypokalemia is an indication that patients are at risk for edema due to Pseudo-Bartter’s syndrome following cessation of purging [9]. Severe metabolic alkalosis (bicarbonate levels > 40 mmol/L) is usually due to vomiting, whereas in laxative use hyperchloremic metabolic acidosis is often seen [41] although after significant volume depletion patients may develop metabolic alkalosis [42].

In addition to differences in these serum values, urinary electrolytes may help elucidate mode of purging. In the case of laxative use leading to diarrhea, urinary sodium is low due to increased renal absorption of sodium. In the case of vomiting, urinary sodium may be decreased or normal due to metabolic alkalosis and increased urinary bicarbonate which binds urinary sodium; however, urinary chloride will be low which can help to distinguish between laxative use and purging. Low urinary chloride may also be seen in diuretic use. High urinary chloride can be seen in gastrointestinal losses from diarrhea when it causes decreased circulating volume leading to excretion of excess acid and ammonium chloride [43]. In the case of diuretic use, urinary sodium will be high as diuretics cause urinary losses of sodium [10]. A summary of the common electrolyte findings in serum and urine is found in Table 1.

**Table 1:** Common electrolyte findings based on mode of purging

Mode of purging	Potassium levels	Sodium levels	Bicarbonate levels	Urinary Na	Urinary Cl
Self-induced vomiting	Decreased	Decreased or normal	Increased	Decreased or normal	Decreased
Laxative abuse	Decreased	Decreased or normal	Increased or normal	Decreased	Increased
Diuretic abuse	Decreased	Decreased or normal	Increased	Increased	Decreased

### Hypomagnesemia

Magnesium is an important component of cardiac and neurologic function [44] and is associated with fatal cardiac arrhythmias and refractory hypokalemia [5]. While there are few studies investigating hypomagnesemia in ED [44], hypomagnesemia is seen when total body stores of magnesium are severely low [7], such as in ED and malnutrition. One older study of patients admitted to an ED unit found 25% of patients had hypomagnesemia [45] Heart failure in a 15 year old girl with AN has been attributed to hypomagnesemia [44]. Hypomagnesemia can be caused by decreased oral intake, diarrhea or diuretic use [5]. Hypomagnesemia is a potential complication of refeeding syndrome as it shifts intracellularly with glucose [7] and thus should thus be monitored closely [30]. A retrospective chart review of hospitalized adolescents with eating disorders including AN (subtype not specified), BN or eating disorder not otherwise specified found approximately 16% of patients developed hypomagnesemia, an average of 4.9 days but as late as 34 after refeeding [44]. Characteristics of patients who were more likely to have hypomagnesemia included longer duration of illness, older age, and increased likelihood of purging [44]. For symptomatic, severe hypomagnesemia (magnesium < 1.25 mg/dL), 1 to 2 g of intravenous magnesium can be administered over an hour. Levels should be rechecked as intracellular repletion takes longer [46]. For milder hypomagnesemia oral repletion can be started at 400–800 mg twice a day.

### Hypophosphatemia

Phosphate is a ubiquitous molecular element that plays essential roles in normal cell functioning, serving as an energy source as a component of adenosine triphosphate (ATP) and a regulator of molecular activity [47]. Hypophosphatemia can cause significant muscle weakness, rhabdomyolysis, and in extreme cases fatal cardiorespiratory failure due to impaired contractility of the myocardium and respiratory muscles [5]. It also has effects on the central nervous system, causing altered mental state, irritability, numbness, or seizures due to its effect on ATP depletion [47]. Prolonged hypophosphatemia affects bone health as it leads to impairment of bone mineralization [47]. Clinical symptoms of hypophosphatemia are widely variable, ranging from asymptomatic to severe symptoms including organ dysfunction and even death; increased severity of symptoms is usually seen at levels < 1 mg/dL [48].

Hypophosphatemia can be found in ED patients prior to and as a complication of refeeding syndrome. Hypophosphatemia prior to refeeding is often related to decreased nutritional intake, and thus is more often found in patients with AN-R [5]. In inpatient and residential patients with ED, 6% of AN (subtype not specified) patients have been found to have hypophosphatemia on admission, which is a predictor of hypophosphatemia during refeeding [9]. Another study of patients admitted with IBW < 65% found 23% of patients on admission had low phosphorus [29].

Hypophosphatemia is the most common complication from refeeding in patients with AN [31]. One study found 35% of patients hospitalized for severe AN (subtype not specified) developed refeeding hypophosphatemia, and 47% of those had critical hypophosphatemia (serum phosphorus < 0.71 mmol/L) [29]. Patients most at risk for hypophosphatemia during refeeding are likely those with IBW < 70% or have had rapid weight loss [31]. Treating hypophosphatemia during refeeding may require thousands of milligrams of oral phosphorous and for levels less than 2 mg/dL, patients should be treated inpatient with intravenous [49] in addition to oral phosphorus.

### Pseudo-Bartter’s syndrome

Pseudo-Bartter’s syndrome refers to complex physiologic changes that occur when volume depletion due to purging activates the renin–angiotensin–aldosterone axis, leading to increased aldosterone secretion at the distal renal tubule [50]. Prostaglandin secretion due to decreased chloride and volume loss perpetuates sodium and chloride loss and renin and aldosterone secretion [50]. These changes can lead to edema formation and rapid weight gain upon abrupt cessation of purging as aldosterone remains elevated for several weeks and thus continues to act on the kidneys [51]. In fact, patients with Pseudo-Bartter’s syndrome can gain up to 10 pounds in the first few days after purging cessation even if they are not given intravenous fluids [52]. Pseudo-Bartter’s syndrome is reversible and can be treated [51] by correcting volume loss with judicious use of intravenous fluids to stop the excess aldosterone secretion, repletion of potassium, and use of an aldosterone antagonist such as spironolactone [50]. Spironolactone should be continued for around 3 weeks, as this is the estimated duration of increased aldosterone secretion from the adrenal glands [51]. Intravenous fluid repletion, if given too rapidly, can significantly worsen edema formation given the high levels of aldosterone leading to avid renal sodium reabsorption [51]. Addressing the edema is important, as it is particularly distressing to patients undergoing treatment for eating disorders to be faced with a significant degree of fluid retention and rapid weight gain while attempting to stop purging behaviors [51].

## Conclusions

ED are psychiatric disorders with significant risk of medical complications, including renal complications, which can include AKI, CKD, electrolyte disturbances, and acid–base disorders. Renal disease, including ESRD requiring dialysis, can occur in ED but is often undiagnosed. Patients with AN and BN should be monitored via routine labs with previously discussed limitations of serum creatinine values in mind for these disorders. Clinicians should consider similar evaluation in patients with ARFID given several studies demonstrating electrolyte disturbances in this population. Care should be taken when patients are undergoing refeeding to recognize and treat fluid and electrolyte abnormalities that can occur including Pseudo-Bartter’s syndrome. Further studies are needed to evaluate renal dysfunction in ED including ARFID. Educating both patients and providers regarding the potentially severe effects of ED behaviors on renal function, particularly purging, is imperative for early intervention to treat and prevent renal disease associated with ED.

## Data Availability

Not applicable.

## Abbreviations

AAN: Atypical anorexia nervosa
AKI: Acute kidney injury
AN: Anorexia nervosa
AN-BP: Anorexia nervosa-binge purge subtype
AN-R: Anorexia nervosa-restricting subtype
ARFID: Avoidant restrictive food intake disorder
ATP: Adenosine triphosphate
BMD: Bone mineral density
BMI: Body mass index
BN: Bulimia nervosa
CKD: Chronic kidney disease
CPM: Central pontine myelinolysis
ECG: Electrocardiogram
ED: Eating disorder/s
eGFR: Estimated glomerular filtration rate
mGFR: Measured glomerular filtration rate
IBW: Ideal body weight
SIADH: Syndrome of inappropriate anti-diuretic hormone
TIN: Tubulointerstitial nephritis
